# Resource allocation within the National AIDS Control Program of Pakistan: a qualitative assessment of decision maker's opinions

**DOI:** 10.1186/1472-6963-7-11

**Published:** 2007-01-23

**Authors:** Sara Husain, Masood Kadir, Zafar Fatmi

**Affiliations:** 1 Health Systems Division, Department of Community Health Sciences, Aga Khan University, Pakistan; 2 Public Health Division, Department of Community Health Sciences, Aga Khan University, Pakistan

## Abstract

**Background:**

Limited resources, whether public or private, demand prioritisation among competing needs to maximise productivity. With a substantial increase in the number of reported HIV cases, little work has been done to understand how resources have been distributed and what factors may have influenced allocation within the newly introduced Enhanced National AIDS Control Program of Pakistan. The objective of this study was to identify perceptions of decision makers about the process of resource allocation within Pakistan's Enhanced National AIDS Control Program.

**Methods:**

A qualitative study was undertaken and in-depth interviews of decision makers at provincial and federal levels responsible to allocate resources within the program were conducted.

**Results:**

HIV was not considered a priority issue by all study participants and external funding for the program was thought to have been accepted because of poor foreign currency reserves and donor agency influence rather than local need. Political influences from the federal government and donor agencies were thought to manipulate distribution of funds within the program. These influences were thought to occur despite the existence of a well-laid out procedure to determine allocation of public resources. Lack of collaboration among departments involved in decision making, a pervasive lack of technical expertise, paucity of information and an atmosphere of *ad hoc *decision making were thought to reduce resistance to external pressures.

**Conclusion:**

Development of a unified program vision through a consultative process and advocacy is necessary to understand goals to be achieved, to enhance program ownership and develop consensus about how money and effort should be directed. Enhancing public sector expertise in planning and budgeting is essential not just for the program, but also to reduce reliance on external agencies for technical support. Strengthening available databases for effective decision making is required to make financial allocations based on real, rather than perceived needs. With a large part of HIV program funding dedicated to public-private partnerships, it becomes imperative to develop public sector capacity to administer contracts, coordinate and monitor activities of the non-governmental sector.

## Background

Public sector funding, like any resource, is limited[1]; and competition exists among various sectors for these limited funds. Countries with different health care systems and different levels of health care spending are struggling to meet increasing health care demands within constrained resources. This makes prioritisation among services an essential activity to determine allocation of resources.

Despite development of decision making tools to assist policy makers in reaching unequivocal and optimal solutions for resource distribution [2-4], the largely subjective technique of incrementalism [5-7] is relied upon instead. Through this method, allocation is determined by expenditures/allocations of the previous year, after making provisions for inflation. Limited use of decision making tools results from a lack of technical knowledge and experience among decision makers[6], and the information intensive nature of tools themselves. Additionally, these tools are normative, focusing exclusively on technical aspects while completely overlooking the complexities of decision-making. These tools assume decision makers are neutral agents, with little or no personal incentives, values and external influences guiding their decisions[3,8].

While calls to make prioritisation within the health care setting more rational and evidence-based exist[9], individual perceptions, values and experiences lend to variable definitions of 'health', and 'need'. Actual allocation is thus a result of bargaining and negotiations among different lobbies and different decision makers. This has been described by Lomas[10] as an interplay between institutional structures for decision making, values embodied within institutions and personnel, along with information and popular opinion. Hence, allocation of resources is not a value free exercise but is intricately linked with needs assessment, political pressures and even donor wishes[8,11]. Thus, along with developing decision making tools, the process of allocating resources and factors influencing these processes need to be focused to identify gaps and suggest improvements in the system of resource allocation[3,8].

### Allocation of resources in the Pakistani context

In Pakistan, the portfolio of Health is a provincial mandate, with federal policies providing guidance[12]. Proposal of a new scheme requires submission of a pre-designed proforma, the Planning Commission Document 1 (PC1) by the Ministry of Health to the Planning Commission. Based on feasibility studies, this document provides information about the proposed project, its activities, and required annual funding. Prior to approval, the submitted document is assessed for technical and financial feasibility by members of the Planning Commission, and Ministries of Finance and Health[12]. This provides a forum for the involved departments to debate about relative merits and demerits of the proposed program and the financial requirements. Final approval of projects is granted at provincial or federal levels depending on the monetary value of the proposed project [13-15].

Though efficient resource utilization is a key public health issue, there is insufficient local literature and perspectives of decision makers about the process remain unknown. The objective of this study was to identify perceptions of decision makers about the process of resource allocation within the Enhanced National AIDS Control Program. The program was chosen because current increases in HIV seroprevalence within the high-risk population and recent investments made to this program, both served to increased its public health importance. Despite these developments, influences determining the allocation of resources within the program have not been studied.

### The HIV/AIDS epidemic and Pakistan's response

First reported two decades ago[16,17], HIV currently ranks as the third leading cause of death due to an infectious agent[18], having caused nearly 3 million deaths during the year 2003 alone[19]. The first case of HIV in the densely populated country of Pakistan was diagnosed in 1987 and till as late as June 2003 only 2,086 cases of HIV/AIDS were officially reported[20]. Although no large-scale surveillance data is currently available, recent data suggests a transition is underway, with the country moving from the stage of low prevalence to a concentrated epidemic[21]. High risk behaviour practices combined with poor knowledge about the disease and its transmission [22-24], generalized poverty and an atmosphere of denial and secrecy [25-27], all present an enormous potential for further viral spread within the general population.

In 1987, the government established the National AIDS Prevention and Control Program (NACP) [28,29], with resources concentrated on establishment of laboratory-based services. In 1993, the NACP was extended; Provincial Implementation Units (PIUs) were established and assigned the task of developing Provincial AIDS Control Programs[30]. Although various strategies were devised to combat the spread of HIV, e.g. screening of blood, mass media campaigns and establishment of surveillance centers, these activities were discordant[28,31]. The National HIV/AIDS Prevention and Control Program of the Government of Pakistan was transformed into the Enhanced National AIDS Control Program with approval of the PC1 in July 2002. This is an Umbrella Project with federal and provincial program components drawn together to combat the spread of HIV in the country.

Globally, HIV/AIDS has received much political and financial commitment[32], with Official Development Assistance (ODA) and private foundations contributing nearly $4.7 billion in 2001, compared to an annual collection of $400 million in 1998[33]. The National AIDS Control Program in Pakistan has also witnessed increased funding since its inception, with a nearly 140% increase in funding of the program spanning the decade from 1991 to 2004. This rapid increase in funding of the program relative to the planned expenditure of the Ministry of Health is explained by a rise in donor funding within the program[34], leading to an increase from 0.01%–0.08% in the first decade to a current 0.29%. This has been seen in other countries where largest planned expenditures relative to the Ministries of Health were in programs receiving donor funding[35]. Despite this rise in donor funding and increasing allocations within national programs, there exists considerable shortfall of external funds[36] and a limited generation of local resources, making efficient allocation of finances necessary.

## Methods

Qualitative methodology was applied to develop an understanding of issues related to resource allocation. Persons considered most informed were decision makers involved in the process of allocating resources within the NACP. Study participants thus represented the Ministries of Health (MOH) and Finance (MOF) and the Planning Commission (PC) at both provincial and federal levels. All study participants were working in the public sector for more than five years and were posted in these departments during 2001–2002, the time during which PC1 for the Enhanced National AIDS Control Program was developed and approved. Changes in two provincial chapters of the National AIDS Control Program resulted in vacancies at the Program Manager level. These posts were vacant for two years prior to the study and remained unfilled at the time of the study. The study was thus conducted in two of four provinces in the country. Selection of participants for interviewing involved identifying the current program managers at federal and provincial levels. Once interviewed, these participants were asked to provide information about other decision makers involved in the concerned ministries who were involved in the development of the PC-1 document. The ministries were then approached and the identified persons interviewed. This technique of contact tracing to identify study participants was done at both provincial and federal levels. No study participant refused an interview, but some interviews required two to three attempts at contacting the officials before the interview could be conducted.

The study was conducted after obtaining approval from The Aga Khan University Ethical Review Committee. An interview guide was developed and pre-tested on two persons, one working in an HIV/AIDS related field, and the other working in the field of policy development. Themes touched upon in the interview guide included participant's understanding of the national process of resource allocation, strengths and gaps working in the system; process of prioritisation and principles for resource allocation within the National AIDS Control Program and influences working on programmatic allocation of resources. The initial interview guide was modified and this modified, pre-tested interview guide was used to conduct ten in-depth interviews. All interviews were conducted by the first author in June and July 2004. During the course of the study, participant responses were incorporated in the interview guide after each interview. These modifications did not add new questions to the interview guide, but allowed for development of better probes enabling detailed discussions with subsequent participants.

The duration of each interview was about 2 hours. Prior to conducting each interview, written informed consent was sought from each participant and written notes were made during all interviews, with an exact transcription of participant responses to questions being noted during the interviews. Most participants (seven in number) refused an audio-recording of the interview, thus increasing reliance on interview notes. Immediately after the interviews, notes were supplemented with observation notes. Recorded interviews were transcribed using MS Word, with notes used to supplement and validate participant responses. Notes of interviews, which were not audio-taped, were similarly transcribed.

All transcripts were analysed using QSR NVivo 5 software, which allows management of qualitative data. Using the transcripts, participant responses to individual questions were separated into themes, with constant reference being made to interview and observation notes. All transcription and analyses were done by the first author. Once all participant responses to individual questions were separated into different themes, further analysis was done to collapse themes by linking together similar experiences and participant responses. This process continued till no new themes or codes could be generated from the collected data. Confidentiality was maintained by restricting access to interview notes and analysing data using a list of pseudonyms.

## Results

Of the ten participants, three represented the Federal government; one each from the departments of Health, Planning and Finance. Of the seven provincial level participants, one represented Finance and three belonged to Health and Planning Divisions of each province. The themes identified through interview analysis are described below, beginning with a description of the process of resource allocation within the public sector to factors thought to influence the process.

### Resource allocation in the public sector

All study participants agreed that the NACP PC1 was approved through due process, as required by the Planning Commission of Pakistan.(13). An approximately Rs 2859 million proposal (US$ 47.538 million), it received approval by the highest economic authority in the country, the Executive Committee of National Economic Council (ECNEC).

The Departments of Finance and Planning have well-defined roles for PC1 evaluation; with technical and costing assessments by the Planning Department, and Finance looking into long-term financial feasibility of the proposal.

The system for allocating resources, was thought to be "highly bureaucratic, tending to become a matter of routine" by study participants at both federal and provincial levels. This tended to "drag down the program", especially if the PC1 document required revisions. The sponsoring agency having no authority to approve adjustments has to reapply with a revised PC1. This reinitiates the entire process of developing and evaluating the revised PC1 thereby causing delays in program initiation and attainment of program targets.

Appraisal by trained economists in the Planning Division ideally requires "quantifications focusing on tangible project results", however if data is deemed insufficient then "(the) experience of working in the public sector" is relied upon. The role of Finance was described as being "restricted to identifying multiplicity of program activities and determining burden of recurrent cost within the proposed projects". As explained by a Federal Finance representative, this was because

"...feasibility of programs in health cannot be done in rupee amounts and is very different from other sectors. As this is technical work, the decision of how much should be allocated to prevention and how much to be kept for cure is that of the implementing agency. We in finance look at the whole envelope and not at individual slots under which allocation is made."

The sponsoring department is responsible for initiating the planning process and negotiate the funding required; the health department however was described by a provincial planning department participant as having a "down stream role" with limited powers of negotiation. This was attributed to a relatively low status of the Ministry of Health compared to more economically productive sectors.

### Influences on resource allocation

Factors ranging from personal to global were described by study participants to influence the division of resources.

**Individual motivation and political pressures **were thought to engender prioritisation of personal gain over national interests among decision makers. One respondent described "resource allocation is a political decision"; where a limited supply of resources and a plethora of demands cause "political pressures and elitism (to) influence(s) decisions at every level, in every department." Another participant felt that although there is an established system of information based priority setting, political influences tend to "spoil this system." These influences manifest as staff reassignments; unnecessary delays in project assessments; approval by persons uninvolved in initial assessment phases and who may lack technical knowledge of the issue. Political pressure exerted to expedite the review process resulting in less time for critical reviews and discussions of project proposals was another gap.

Priority setting for the NACP was described a Federal level exercise, with the degree of provincial involvement in framing policies a "mere cosmetic process". Development of the policy document for the NACP, the National Strategic Framework, had invitations based on "personal choices at the federal level rather than technical merit of candidates". Rural representation was deemed insufficient with an overwhelming dominance of donor agencies in the forum. Federal influence during formulation and approval of the NACP PC 1 document, in staffing and financing decisions existed, as claimed by all study participants at the provincial level. Exerted through a system of reward and punishment; this influence was described by a participant as

"sidelining the person who talks a lot and issue(ing) letters to highlight problems, while the persons liked are repeatedly nominated for foreign trainings and trips."

Provincial Finance and Planning departments' involvement in developing provincial PC 1s were described by two participants as "(A) 2–3 day negotiation process", with limited consideration to individual program capacities and needs as the PC1s were based on federally developed templates. This claim of limited involvement of the provincial departments was refuted by the Federal program, which declared provincial governments and programs had complete autonomy in developing the PC1 with the federal program providing "guidance, not interruptions".

Most study participants considered frequent transfers within the bureaucracy as cause of discontent and apathy within the workforce. One participant described it a "loss of institutional memory and experience", resulting in decision-making positions being filled on basis of experience, not expertise. Lack of technical knowledge within the public sector was also thought to result from a failure to attract proficient personnel owing to low public salary structures. As achievements were considered "highly person-dependent", program management capacity was unequivocally accepted as essential for program success.

Retention of trainings and workshops, (soft program elements) along with their associated grants, with transference of loans to the provinces was considered by four provincial participants as Federal influence. The federal program rationalized retention of the Second Generation Surveillance system (funded through CIDA grant component) to enable coordination among the provincial cells. Workshops for migrant workers and educational activities targeting the youth were retained because of the centralized functioning of the collaborating line ministries i.e. Bureau of Immigration and Ministry of Education.

**Donor agency influence **in the development and approval of the PC1 document of the NACP was considerable, as reported by all, but one study participant. This was thought to be a result of limited provincial level data, paucity of trained manpower and non-cooperation among the involved departments. Another suggested reason was the "need to improve balance of payments within the country without consideration to technical aspects of the project", an issue that "could not be fully appreciated at the provincial level". A federally placed participant revealed that despite the existence in the public sector of criteria determining project selection, those projects which received foreign funding, particularly grants topped the priority list for funding and approval.

"There was a pressure to ignore all things ...it was a political decision to improve the exchange reserves for social and economic benefit."

The initial reaction of provincial governments, as revealed by four participants, was a rejection of the program because HIV was not considered a Public Health threat and loans for the program were transferable to the provincial governments. The increased interest in the issue was perceived to be a demonstration of global trends and donor agency influence on local policies, rather than a real need. A provincial planning division representative thought that though AIDS is an issue, the government should not be "blind to real threats (of) diseases like Hepatitis B and C", which are "larger Public Health issues for the country (and) are not receiving international funding and are mostly locally funded".

This donor influence was thought to be promoted by "sympathetic agents of interest holding public office", incentives of foreign visits and threats of withdrawing financial assistance altogether. Little rationale could be attributed for such influence other than "donors have their own agenda", and one respondent describing it "(a) tool of colonisation".

Four participants at both federal and provincial levels considered the strategy of public-private partnership adopted in the program to be "donor promoted jargon" and shared their concerns in involving NGOs at a national level. One participant felt "the social value attached to the program (NACP) can only be given by the public sector". The costing exercise for the PC1 was described by a provincial program manager as donor driven, with little or no input of local data. This data provided by donor agencies was suspected of being "biased and reflective of their own agenda rather than ground realities". A senior official at the Federal program level however, refuted this claim stating donors were responsive to local program requirements and demands, as evidenced in the National Strategic Framework. Only one participant at the provincial level spoke of the inclusion of Hepatitis B and C testing kits in the PC1 of the NACP as a "triumph for the government in persuading (donor agencies) to agree to funding this activity".

**Informed decision-making **though acknowledged by all participants as being essential for efficient resource allocation, was not being employed owing to a lack of knowledge about the local situation and an existing norm of not basing decisions on evidence. The lack of a standardised system to determine allocation and "program novelty of the NACP" reduced the activity to "a largely subjective exercise, directed by gut-feeling". The federal program refuted this claim, alleging use of costing exercises and scientific data to determine allocation.

Study participants supported the importance of generating local data, given a unique local socio-cultural and demographic context in which international data may not find an application. Participants at the provincial level however, claimed international literature and donor-provided data was utilized owing to a lack of valid local studies, their limited dissemination to and use by policy makers. This view contrasted with representatives of the federal government who claimed development of PC1 was based on local studies outsourced by the NACP.

All participants at the provincial level and those representing the federal finance and planning departments denied use of any economic tools e.g. cost-benefit/effectiveness analysis or models for allocating resources within the NACP. In fact, most were unaware of the existence of this decision making tool. Use of such information was described as being at a "very primitive stage". They were not employed even in the planning department, which is a "support department". The federal program however reported using "resource need" models to determine allocation.

All participants, except one considered public opinion to exert negligible influence in determining priorities and allocation, attributing it to a low priority accorded to health and poor involvement of the public in decision-making processes. Similarly, influence of private sector physicians and physician bodies was limited as a result of poor interest in preventive activities and a limited influence of professional bodies on priority setting.

Seven participants thought the principles of equity and efficiency to be "slogans and jargon" that are ignored when allocating resources. This culture was pervasive, be it at the level of the country or at departmental or even programmatic levels. Though the vision guiding activities within the health sector is 'Health for All', participants were not able to describe how this vision was translated into reality through an allocation of resources. As a federally placed participant described

"Lack of equity is visible within the macro-financing framework of the country, where a mere US $4 per capita expenditure is on health. This inequity is visible within the health sector as well, with more money being spent on hospitals and tertiary care than on primary care."

One provincial program manager however, felt allocation within the NACP had addressed equity and efficiency through planning of activities for both general and high risk populations.

**The dilemma of allocating resources **to the NACP was the distribution of limited funds to a disease considered "irreligious and immoral" by a large conservative section of the society; as expressed by participants not belonging to the program.

"The cake is small and there are many mouths e.g. should we be putting money in the HIV/AIDS program or should we provide money to provide safe water to people, given that diarrhoea is still the leading cause of death in our country."

Differences of opinion existed regarding provision of anti-retroviral medications. Program management and federal level participants felt that lack of treatment incentive may be a potential hurdle in program expansion and acceptability. Other provincial level decision makers tended to favor preventive activities, describing high treatment costs and a tendency to emphasize numbers in output as obstacles.

*"If 10 people can be treated versus a single AIDS patient, then numbers will be opted for (as apposed to treatment for infected persons)"*.

The existence of the NACP and a "ten-fold increase in the allocated resources" was considered an indication of governmental commitment to address the issue of HIV in the country. However, provincial planning and finance division participants did not consider HIV to be a Public Health issue owing to the small number of diagnosed cases in the country.

## Discussion

The study sought to identify issues that influence allocation of resources within public health programs, a little studied phenomenon in local literature. As the new phase of the NACP began in 2002, experiences of decision makers are relatively recent and less likely to suffer a loss of recall. To gain a variety of perspectives and opinions, interviews of decision makers at various levels and in different departments within the bureaucracy were conducted. The participants selected for interviewing were those who are required to provide the most amount of technical input at the time of developing the PC1. Though several decision makers within the public sector hierarchy are involved, most are not expected to provide technical input to develop the PC1 and make decisions on the proposed PC1s instead. Inclusion of such public officials from different provinces and other stakeholders may have provided greater insight into the process of resource allocation.

Although Pakistan has a low reported prevalence of HIV, there exists an enormous potential for viral spread. Given this potential, implementation of comprehensive strategies including preventive activities, surveillance systems to detect cases early and provision of treatment and care for HIV positive individual becomes imperative[37,38]. These are all present in the current Enhanced National AIDS Control Program. There are however, operational and financing aspects that distinguish this program from other healthcare programs within the public sector. These include extensive donor funding and public-private partnerships, with NGOs serving as primary service providers within the program.

Study participants considered the existence of an unambiguous hierarchical system of allocating public money, with well-described responsibilities, authorities and a separation of power at each level of decision-making an asset. The effective functioning of this system however, suffers owing to a paucity of information, lack of trained manpower and political and bureaucratic influence; issues identified in other studies focusing on the decision making structure within the country[7,14,39]. This was thought to result in allocation of resources through incremental budgeting and gut feeling with limited, if any use being made of cost-effectiveness data, scientific literature and public opinion[7,40]. Virtual non-use of decision making tools to determine optimal allocation resulted from a poor understanding and knowledge of the utility of such tools, along with limited expertise in efficient budgeting. This increased reliance of the system on external assistance and expertise, thereby exposing the system to pre-determined priorities and limiting the autonomy of local decision makers[35].

An enrichment of public sector expertise in planning and budgeting through staff trainings and attracting technical expertise by revising public pay scales is essential to make decision making more rational, evidence-based and justifiable, and to reduce dependency on external agencies for technical support. Lack of valid information, considered an obstacle by most decision makers, identifies a need for strengthening both the public health database (Health Management Information System) and research by the local scientific community. A success story that may be an important lesson for the health system is the effective persuasion by decision makers to include test kits for Hepatitis B and C in the NACP. An in-depth analysis of factors that resulted in this relative success could serve to pinpoint strengths within the system that could be enhanced to promote local influences to guide allocation of resources.

Federal level respondents and program managers consider HIV a high priority issue because of its potential for spread and the high cost of neglect associated with the disease. Provincial reluctance to accept the program resulted from an underestimation of disease risk and perceptions that "market influences" rather than local need determined program existence. This difference in perceptions may have resulted from the continuous sponsorship by program managers and donor agencies at the federal level. Federal retention of grant components, though explained to be a result of institutional capacity and bureaucratic jurisdiction, was perceived as interference at the provincial level. These reflect a degree of conflict and distrust existing within the institutional structure. The promulgation of an extensively financed program where partners fail to share program vision may create problems in achieving program targets. To address this concern, it becomes important to develop communications among partner departments and maintain involvement of all actors at every stage of program development and implementation.

The NACP opens new challenges for developing and maintaining public-private partnerships in an atmosphere charged with scepticism. Much of the program funds are being funnelled to NGO-led activities, at a stage when public-private partnership is viewed as a donor driven agenda rather than local need. Although promoted as a means of achieving efficiency in the public health sector by creating competition, it is essential to consider local NGO capacity to deliver the required service. Also, public sector capacity to administer such contracts and effectively coordinate among various NGOs to achieve program goals and targets will need development[41].

Study participants succinctly identified how taboos associated with HIV present a dilemma to decision makers when funding activities. Although the dilemma is not unexpected for a traditionalist society like Pakistan[26,29], it underscores the social responsibility a government has towards its populace, particularly ostracised members of society. Though no simple solutions to these ethical problems exist, broad-based debates with stakeholder involvement to guide resource planning and allocation will sensitise decision makers about local needs, ensure transparency of allocations, thereby resulting in wider support and acceptance.

## Conclusion

The allocation of resources, whether within health or other sectors, is not merely based on technical issues but results from a complex interplay of political, ethical and technical judgements. A lack of consensus regarding which principle should guide the allocation of resources has resulted in calls to make the process of allocation one of "procedural justice"[42]. This requires decision making to be performed by institutions that have legal and constitutional authority, with decisions being made through fair processes that are relevant, defensible and open to public challenges and appeals.

Proper distribution of finances is an essential aspect of planning within any sector, more so in the public sector of Pakistan, given its resource constraints. Though there is an existing institutionalised system to allocate resources through a process of informed debate, the system is not functioning ideally. There is extensive influence of external factors including personal and political influences. Resource allocation within the NACP of Pakistan was thought to have undergone similar influences. Donor influence could be accounted for by its unique nature of being extensively financed through International Development Assistance[34]. Ethical dilemmas arising from public financing of a program providing services focusing on marginalised members of society for a disease thought mostly to spread through illicit and unnatural sexual offences are also unique to the NACP.

To promote efficient allocation of resources in such a system requires making decision makers knowledgeable about HIV, building technical capacity for effective resource allocation, instilling the importance of data based decision making within the institutional set up and ensuring availability of relevant, updated and valid information. Development of consensus enhancing mechanisms ensuring broader participation is essential to enhance ownership, develop a unified vision of action and ensure accountability.

## Competing interests

The author(s) declare that they have no competing interests.

## Authors' contributions

This manuscript is derived from the thesis of the Corresponding Author, SH. SH conceived the idea, developed the study design, collected and analysed data and drafted the document. MK provided technical input in the study design and helped draft the document. ZF provided technical feedback and helped draft the document. All authors read and approved the final manuscript.

## Pre-publication history

The pre-publication history for this paper can be accessed here:



## References

[B1] Samuelson PA, Nordhaus WD, Lucille.H.Sutton ER (1995). Economics.

[B2] Ham C (1997). Priority setting in health care: learning from international experience. Health Policy.

[B3] Holm S (1998). Goodbye to simple solutions: the second phase of priority setting in health care. BMJ.

[B4] Mitton C, Donaldson C (2003). Resource allocation in health care: beyond economics. Health Care Anal.

[B5] Ham C (1993). Priority setting in the NHS: reports from six districts. BMJ.

[B6] Mitton C, Donaldson C (2002). Setting priorities in Canadian regional health authorities: a survey of key decision makers. Health Policy.

[B7] Green A, Ali B, Naeem A, Ross D (2000). Resource allocation and budgetary mechanisms for decentralized health systems: experiences from Baluchistan, Pakistan. Bull World Health Organ.

[B8] Klein R (1993). Dimensions of rationing: who should do what?. BMJ.

[B9] Hoedemaekers R, Dekkers W (2003). Key concepts in health care priority setting. Health Care Anal.

[B10] Lomas J (2000). Connecting research and policy. Canadian Journal of Policy Research.

[B11] Khan A (1996). Policy-making in Pakistan's population programme. Health Policy Plan.

[B12] Ghaffar A, Kazi BM, Salman M (2000). Health care systems in transition III. Pakistan, Part I. An overview of the health care system in Pakistan. J Public Health Med.

[B13] Khattak FH (1996). Financing of health sector. Health economics and planning in Pakistan.

[B14] (2003). District Planning Manual.

[B15] Kemal AR (2001). Who makes economic policies? The players behind the scene.

[B16] Gibney L, DiClemente RJ, Vermund SH (1999). Preventing HIV in Developing Countries: Biomedical and Behavioral Approaches.

[B17] Sepkowitz KA (2001). AIDS--the first 20 years. N Engl J Med.

[B18] (2002). Reducing Risks, Promoting Healthy Lifestyles. The World Health Report.

[B19] (2003). AIDS epidemic update.

[B20] Shah SA (2004). Epidemiology of HIV/AIDS: Karachi..

[B21] Shah SA, Altaf A, Mujeeb SA, Memon A (2006). An outbreak of HIV infection among injection drug users in a small town of Pakistan: potential for national implications. J Pak Med Assoc.

[B22] Najmi R (1998). Awareness of health care personnel about preventive aspects of HIV infection/AIDS and their practices and attitudes concerning such patients.. J Pak Med Assoc.

[B23] Sheikh NS, Sheikh AS, Shan RU, Sheikh AA (2003). Awareness of HIV and AIDS among fishermen in coastal areas of Balochistan. J Coll Physicians Surg Pak.

[B24] Sikandar QM, Malik R, Afzal R (2000). Knowledge, attitude and practices of college students of Rawalpindi regarding HIV/AIDS. Pak J Med Res.

[B25] Afsar HA, Mahmood MA, Barney N, Ali S, Kadir MM, Bilgrami M (2002). Community knowledge, attitude and practices regarding sexually transmitted infections in a rural district of Pakistan. J Pak Med Assoc.

[B26] Iqbal N Health-Pakistan: Taboos Still Hamper AIDS Programme. http://www.aegis.com/news/ips/2001/IP010407.html.

[B27] Qidwai W (2002). Knowledge about sexually transmitted infections among young Pakistani men. J Pak Med Assoc.

[B28] Shah SA, Khanani MR, Mujeeb SA, Luby S, Ali S, Memon A, Baqi S (1998). AIDS in Pakistan.

[B29] Hyder AA, Khan OA, Shah SA, Memon MA, Khanani MR, Ali S (1999). Sub-national response in HIV/AIDS: a case study in AIDS prevention and control from Sindh province, Pakistan. Public Health.

[B30] Kazi BM, Ghaffar A, Salman M (2000). Health care systems in transition III. Pakistan, Part II. Pakistan's response to HIV-AIDS. Journal of Public Health Medicine.

[B31] Khan OA, Hyder AA (2001). Responses to an emerging threat: HIV/AIDS policy in Pakistan. Health Policy Plan.

[B32] (2001). The Global Strategy Framework on HIV/AIDS.

[B33] UK's Call for Action on HIV/AIDS. http://www.dfid.gov.uk/pubs/files/aidscallforaction.pdf.

[B34] Public Sector Development Program PC (1991). Program for Prevention of AIDS Diseases in Pakistan, National Institute of Health.

[B35] Bollinger L, Stover J (2000). How do AIDS Control Program Managers make resource allocation decisions?.

[B36] Schwartländer B, Stover J, Walker N, Bollinger L, Gutierrez JP, McGreevey W, Opuni M, Forsythe S, Kumaranayake L, Watts C, Bertozzi S Resource Needs for HIV/AIDS. http://www.sciencexpress.org/21June2001/Page1/10.1126/science.1062876.

[B37] Mukherjee JS, Farmer PE, Niyizonkiza D, McCorkle L, Vanderwarker C, Teixeira P, Kim JY (2003). Tackling HIV in resource poor countries. BMJ.

[B38] Kitahata MM, Tegger MK, Wagner EH, Holmes KK (2002). Comprehensive health care for people infected with HIV in developing countries. BMJ.

[B39] Green A, Rana M, Ross D, Thunhurst C (1997). Health planning in Pakistan: a case study. Int J Health Plann Manage.

[B40] Green A, Ali B, Naeem A, Vassall A (2001). Using costing as a district planning and management tool in Balochistan, Pakistan. Health Policy Plan.

[B41] Mills A (1998). To contract or not to contract? Issues for low and middle income countries. Health Policy Plan.

[B42] Gibson JL, Martin DK, Singer PA (2002). Priority setting for new technologies in medicine: a transdisciplinary study. BMC Health Serv Res.

